# The cerebellum computes frequency dynamics for motions with numerical precision and cross-individual uniformity

**DOI:** 10.21203/rs.3.rs-4615547/v1

**Published:** 2024-07-30

**Authors:** Chia-Wei Liu, Shun-Ying Chen, Yi-Mei Wang, Liang-Yin Lu, Peng Chen, Ting-Yu Liang, Wen-Chuan Liu, Ami Kumar, Sheng-Han Kuo, Jye-Chang Lee, Chung-Chuan Lo, Shun-Chi Wu, Ming-Kai Pan

**Affiliations:** 1 Department and Graduate Institute of Pharmacology, National Taiwan University College of Medicine, Taipei, Taiwan.; 2 Molecular Imaging Center, National Taiwan University, Taipei, Taiwan.; 3 Institute of Biomedical Sciences, Academia Sinica, Taipei, Taiwan; 4 Cerebellar Research Center, National Taiwan University Hospital, Yun-Lin Branch, Yun-Lin, Taiwan; 5 Department of Medical Research, National Taiwan University Hospital, Taipei, Taiwan; 6 The Initiative for Columbia Ataxia and Tremor, New York, NY, USA; 7 Department of Neurology, Columbia University, New York, NY, USA.; 8 Institute of Systems Neuroscience, National Chin-Hua University, Shin-Chu, Taiwan.; 9 Department of Engineering and Bioinformatics, Chin-Hua University, Shin-Chu, Taiwan.

**Keywords:** Motor kinematics, Motor control, Cerebellum, Frequency, Neuronal coding, Oscillations, Electroencephalogram

## Abstract

Cross-individual variability is considered the essence of biology, preventing precise mathematical descriptions of biological motion^1–7^ like the physics law of motion. Here we report that the cerebellum shapes motor kinematics by encoding dynamic motor frequencies with remarkable numerical precision and cross-individual uniformity. Using in-vivo electrophysiology and optogenetics in mice, we confirmed that deep cerebellar neurons encoded frequencies via populational tuning of neuronal firing probabilities, creating cerebellar oscillations and motions with matched frequencies. The mechanism was consistently presented in self-generated rhythmic and non-rhythmic motions triggered by a vibrational platform, or skilled tongue movements of licking in all tested mice with cross-individual uniformity. The precision and uniformity allowed us to engineer complex motor kinematics with designed frequencies. We further validated the frequency-coding function of the human cerebellum using cerebellar electroencephalography recordings and alternating-current stimulation during voluntary tapping tasks. Our findings reveal a cerebellar algorithm for motor kinematics with precision and uniformity, the mathematical foundation for brain-computer interface for motor control.

## Graphical Abstract

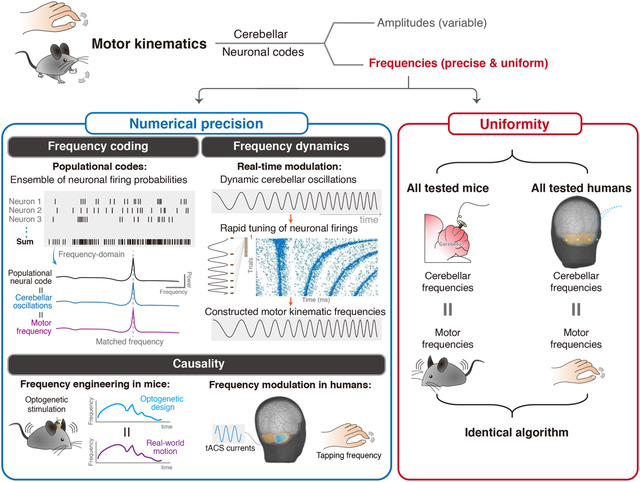

## Introduction

Individual variability has long been a defining yet challenging aspect of biological sciences, distinguishing it from the exact sciences like physics or chemistry. While biological mechanisms are qualitatively valid, calibrating parameters on an individual basis is often necessary. A few notable exceptions, such as the trinucleotide RNA codes for amino-acid translation, have led to significant breakthroughs in biology and medicine. These codes, which are quantitatively precise and universally applicable across cells, have ushered in the era of genetic engineering, gene therapies, and RNA vaccines. However, similar precision and generalizability in the neural dynamics of motor control have yet to be achieved.

Our brains are capable of generating diverse motor behaviors, covering a highly complex set of spatiotemporal kinematic patterns. Although the mechanisms of motor control are often nonlinear and multidimensional, recent studies suggest that the cerebellum plays a crucial role in linearly coding the kinematics. The cerebellum regulates the end-point precision of reach movement^1,2^, motor-state changes of skilled movement^3–5^, eye saccades^6,7^, tongue^8^ and harmaline-induced movements^9^. The cerebellum is also adept at maintaining temporal accuracy^10^, establishing specialized cortical connections^11^ and forming rapid olivocerebellar circuits^12^ for handling fast kinematics. The evidence suggests that our central nervous system may use the cerebellum as a linear encoder to build complex motor kinematics. However, individual variability remains an intrinsic feature of these time-domain observations.

Fortunately, insights into human cerebellar disorders have shed light on the cerebellum’s role in motor kinematics coding. Cerebellar dysfunctions lead to the breakdown of motor kinematic control in a unique feature linked to motor frequencies. Essential tremor, the most common movement disorder, is characterized by involuntary rhythmic movements with a consistent motor frequency, linked to excessive cerebellar oscillations^13–15^. Conversely, cerebellar ataxia features arrhythmic involuntary movements, that are associated with Purkinje cell loss^16,17^. These abnormalities strongly suggest that cerebellar diseases have neuronal coding dysfunctions in forming motor frequencies.

This study investigates the potential of cerebellar frequency coding in shaping motor kinematics. We explored the frequency building blocks at both cellular and population levels and established that motor frequency coding is not only biologically robust but also mathematically precise and generalizable. This suggests a cerebellar algorithm capable of creating complex motor kinematics with designed frequency dynamics.

## Results

### Frequency-dependent cerebellar oscillations precisely report motor rhythms.

**Fig. 1 |:**
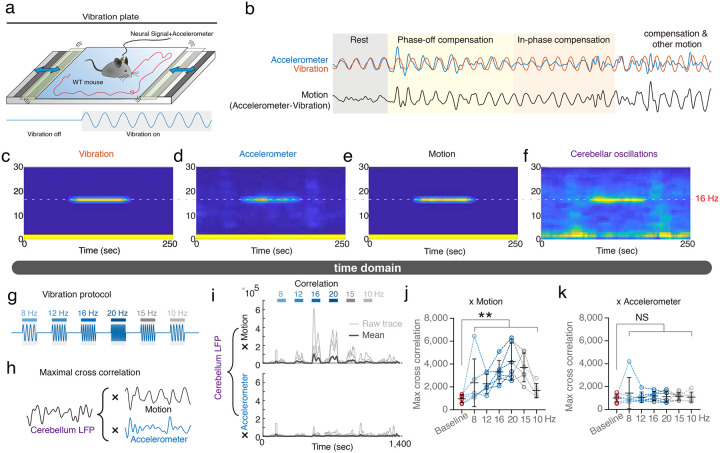
Self-generated cerebellar oscillations in compensatory motions. **(a)** An experimental setting of the vibration platform generating horizontal sinusoidal motions. **(b)** Representative traces for active compensatory motion, calculated as signals of a head-mounted accelerometer minus the platform vibrations. **(c–f)** Representative time-frequency plots of vibrations (c), head-mounted accelerometer signals (d), compensatory motions (e), and cerebellar oscillations (f) during 16-Hz vibrations. **(g)** Schematic of the vibration protocol, indicating the sequence of applied frequencies. **(h)** Illustration of maximal cross-correlation for cerebellar LFPs with compensatory motions and residual body movements (accelerometer). **(i-k)** Trial-by-trial (i) and group analysis (j–k) of cross-correlation from cerebellar oscillations versus compensatory motions or accelerometer signals at various vibrating frequencies. Cerebellar oscillations exhibited significant correlations with compensatory motions (j) but not with residual body movements (k) (n = 6 mice). Error bars denote S.D. **p < 0.01, One-way ANOVA.

Our initial investigations focused on whether the cerebellum encodes the motor frequencies of self-generated rhythmic movements in mice. To trick the mice into generating motor behaviors at a pre-determined frequency, we applied a horizontal vibrating platform that can vibrate at a specific fixed frequency or frequency as a function of time (Fig. 1a and **Video S1**). Wild-type mice were trained to develop active motor compensation to the vibrations and could walk and stand freely on the platform (**Video S2**). Self-generating motion can be calculated by subtracting the pre-designed sinusoidal platform vibrations from the head-mounted accelerometer signals, including both vibration and active motion (Fig. 1b). Both platform and head signals were detected simultaneously with accelerometers of the same design. When the mouse was at rest, the head moved with the platform, leading to similar waveforms of accelerometer signals from the head or the platform (Fig. 1b, **gray part**). When the mouse performed compensatory movement to cancel out the platform vibrations, the head signals were dampened by motor compensation (Fig. 1b, **orange part** and **Video S2**). The vibrations also allowed multiple muscles and joints to react at the same rhythm, which enhanced the frequency information across cerebellar topography. During 16-Hz platform vibrations, simultaneous local field potential (LFP) recordings from the cerebellar cortex revealed corresponding 16-Hz cerebellar oscillations (Fig. 1c–f). Based on this initial observation, we trained the mice with a protocol including multiple vibratory frequencies (Fig. 1g), covering the physiological frequency range of spontaneous motor behaviors^18^.

We first performed a cross-correlation analysis between cerebellar LFPs and mouse motions (Fig. 1h). Compatible with previous knowledge, cerebellar signals are positively and significantly correlated with motions (Fig. 1i–j). However, it is possible that the cerebellum LFPs predominantly reflects the sensory inputs. We therefore cross-correlated cerebellar LFPs with accelerometer signals, which reflected overall motions and therefore corresponding overall sensory inputs. While the accelerometer signals also had strong frequency-dependency (Fig. 1d), they were poorly correlated with cerebellar signals (Fig. 1k), suggesting a motor-predominant contribution of the cerebellar LFPs. While the cerebellar LFPs significantly represent motor kinematics, the cross-correlogram are highly variable across time and across individual mouse (Fig. 1i–j), indicating a qualitative valid and quantitative imprecise scenario.

**Fig. 2 |:**
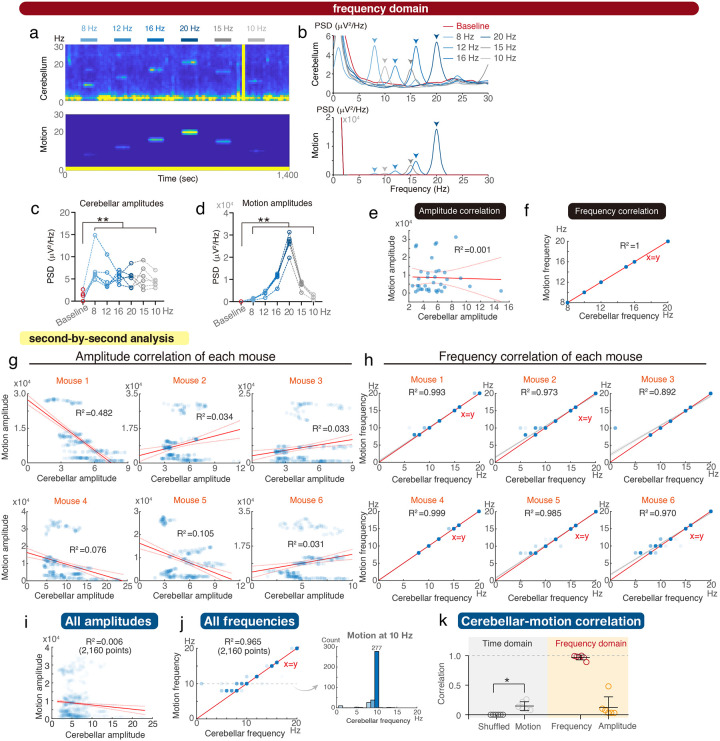
Correlation of cerebellar oscillations and rhythmic motions in the frequency domain. **(a-b)** Representative time-frequency plots (a) and power spectral density (PSD) (b) across various vibrating frequencies. **(c-d)** Peak PSD amplitudes of cerebellar oscillations (c) and compensatory motions (d) across various vibrating frequencies. **(e)** Linear regression analysis of peak PSD amplitudes between cerebellar oscillations and compensatory motor movements. The solid red line represents the best-fit linear model, while the dashed red lines indicate the 95% confidence bounds (36 points in 6 mice). **(f)** Linear regression analysis of the frequencies at peak PSD amplitudes for cerebellar oscillations and motor activities (36 points in 6 mice). **(g-h)** Second-by-second linear regression analysis for each mouse (360 points in each mouse). **(i-j)** Collective second-by-second analysis for all mice combined (2,160 points in 6 mice). **(k)** Statistical analysis of the correlation between cerebellar LFPs and motor activity in both the time domain and frequency domain, using Pearson correlation and the determination coefficient (R^2^) of the linear regression presented in Fig. 2i–j respectively (n = 6 mice). Error bars denote S.D. p* <0.05, **p < 0.01, One-way ANOVA.

Next, we processed the same signals in the frequency domain (Fig. 2a–d). The trained mice consistently produced movements at corresponding motor frequencies, with notably enhanced cerebellar LFP amplitudes (Fig. 2c). However, the enhanced LFP amplitudes were highly variable and did not follow the frequency-dependent increment of motion powers (Fig. 2d), unable to precisely correlated with motor amplitudes (Fig. 2e). In contrast, peak cerebellar oscillatory frequencies accurately encoded motor frequencies, demonstrating minimal individual variability and underscoring the cerebellum’s potential role in quantitative motor-rhythm coding (Fig. 2f).

The extracted frequency in Fig. 2f is the section-based average of frequency-dependent motions. If the cerebellum truly engages in the rhythm control of motor kinematics, the frequency coding should precisely reflect kinematic details. We performed a second-by-second analysis of all recordings, examining frequencies and amplitudes on a second-by-second basis (Fig. 2g–h). The cerebellar frequency consistently matched the motor frequency across all mice and throughout most of the 2,160 data points, highlighting a robust, quantitatively precise coding mechanism (Fig. 2g–j). By comparing the time and frequency domains, the imprecision of cerebellar kinematic coding is mainly contributed by the amplitudes mismatches between cerebellar and motion signals (Fig. 2k). Next, we evaluated the interposed nucleus of the deep cerebellar nuclei (DCN), the output structure of the motor cerebellum. The DCN LFPs were significantly but variably correlated with the motor kinematics in the time domain (**Supplementary Fig. 1**), whereas LFP frequencies consistently matched motor frequencies across all examined mice and all 2,880 data points (**Supplementary Fig. 2**).

In summary, the cerebellum accurately encodes motor frequencies during self-generated rhythmic movements in mice, with minimal observable individual variability.

### DCN neurons calculate motor frequencies throughout populational coding.

**Fig. 3 |:**
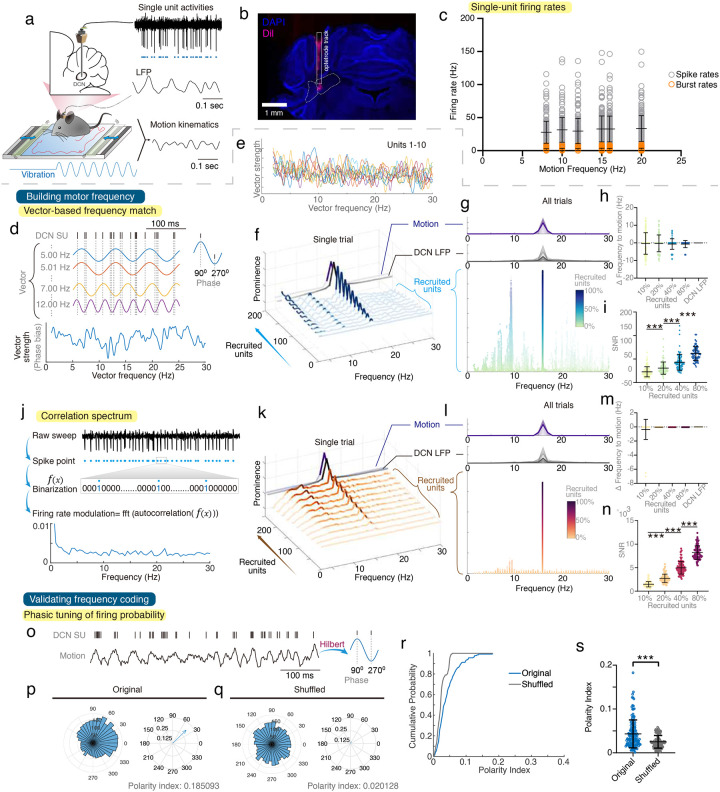
Neuronal coding for rhythmic motions. **(a)** Scheme of simultaneous recordings of single-unit (SU) neuronal activities, DCN LFPs, and motion kinematics. **(b)** A representative plot of the optetrode trajectory labeled with Dil (see Methods). **(c)** SU-firing rates (gray circles) and burst rates (orange circles) in DCN versus motion frequencies (n = 222 units from 8 mice). **(d)** Scheme of the vector strength spectrum analysis. **(e)** Vector strengths of 10 single units. **(f)** Frequency convergence of the vector strength of a representative trial during 16-Hz vibration. The vector strength spectrum peaks converged to the motion frequency throughout the random recruitment of units. Intensity is in arbitrary units of vector strength (no unit), LFPs, or motions (mV). The blue spectrum represents the mean vector strength of recruited units, the black spectrum represents the DCN LFP, and the purple spectrum represents the motion. **(g)** Frequency convergence of motions, LFPs, and vector strengths in all trials. The top two subplots showed the frequency spectrum of motion (top) and cerebellar LFP (middle). Light lines represent single trials, and heavy lines represent the averages of all trials. The bottom figure showed all peaks with sufficient prominence (see methods) detected in the vector strength spectrums throughout the random recruitment of units. The color gradient from green to blue reflected increasing units recruited to calculate the vector strength spectrum. The color depth indicated the level of prominence (n= 138 units from 8 mice. Units with minimum spike number < 10 were excluded to avoid unreliable computation of vector strength). **(h-i)** Quantitative analysis of vector strength spectrums. Peak frequency differences to motions (h) from vector strength spectrum (left four, green to blue) or from DCN LFPs (rightmost, gray), and the signal-to-noise ratio (SNR, Fig. 3i), indicating peak significance of corresponding vector strength spectrums. **(j-n)** The tuning frequencies of neuronal firing probabilities via autocorrelation spectrum (j) with a representative trial (k), group analysis (l), and quantification (m-n). **(o)** Scheme of the phasic tuning of SU firing probabilities to the instantaneous phases of motion. **(p-q)** Representative polar plots. DCN neurons had a greater phasic bias to the phase of motion, quantified by the polarity index. **(r-s)** Group analysis of cumulative probabilities (r) and values (s) of polarity indexes. DCN neurons revealed stronger phasic tuning to 16-Hz compensatory motion at the populational level (n = 138 units from 8 mice). See methods for detailed definitions of burst detection, vector strength, and peak prominence. Error bars denote S.D. ***p < 0.001, One-way ANOVA (i, n), Wilcoxon matched-pairs signed rank test (s).

LFPs are spatiotemporal summation of neuronal signals. We need to understand the building block at the single-cell level. To understand these signals at the single-cell level, we simultaneously recorded single-unit activities and LFPs from the interposed nuclei of the DCN and analyzed corresponding motor kinematics in freely moving mice (Fig. 3a–b and **Supplementary Fig. 3**). We first evaluated whether DCN neuronal firing rates can represent motor frequencies. The motor frequencies were poorly correlated with neuronal firing rates, burst rates, or their mean firing rates (Fig. 3c), against a simple rate-coding algorithm. We next evaluated whether the changes in firing probability, instead of the firing rate itself, could have a tuning periodicity to represent motor frequencies. We leveraged vector strength spectrum analysis^19–23^, a mathematical method using frequency vectors to unbiasedly extract probability tuning strength across frequencies (Fig. 3d). The vector strength frequencies were high variable at the single-cell level (Fig. 3e). However, a specific frequency emerged with improving prominence when more and more neurons were recruited (Fig. 3f). This populationally encoded frequency converged toward the matched DCN oscillatory frequency and motor frequency with the same numerical value (Fig. 3g–h), with increasing signal-to-noise ratio during the recruitment (Fig. 3i). This populational coding mechanism remained valid across all tested frequencies (**Supplementary Fig. 4**). Next, we applied autocorrelation to explore the intrinsic tuning of neuronal firing probabilities (Fig. 3j–n). Similar to the results of vector strength analysis, the autocorrelogram did not generate consistent tuning frequency at the single cell level but faithfully reported the motor frequencies at the populational level (Fig. 3j–n and **Supplementary Fig. 5**).

If the DCN neurons contribute to the generation of motor frequencies, the neuronal firing times should not be random but periodically tuned to the phases of the frequency-dependent motor kinematics. To validate the prediction, we extracted the instantaneous phases of motor kinematics based on the neuronal firing times (Fig. 3o) and quantified the phasic bias by polarity index, a numerical index ranging from 0 (purely random firings) to 1(complete phase-locked firings)^13^. While some units exhibited higher polarity when compared to the shuffled data (Fig. 3p–q), all units have relatively low polarity index (<0.4) (Fig. 3r); therefore, no single neuron can explain the precise frequency coding of motor kinematics. Notably, the neuronal firings have stronger recruitment of higher biased units and greater polarity indexes at the populational level (Fig. 3r–s, **Supplementary Figs. 6–7**). Direct visualization of simultaneously recorded single units also supported the prediction of the abovementioned frequency and phase analysis with populational recruitment (**Supplementary Figs. 8**).

Despite the findings on various tuning frequencies, the average firing rates of DCN neurons remained similar (Fig. 3c). We also performed computational modeling of noisy DCN neurons with the baseline firing rates at 20–22 Hz. When receiving inhibitory inputs of PCs at the frequency of 16 Hz, the populational tuning frequency converged to 16 Hz, while the mean firing rates stayed the same (**Supplementary Fig. 9**). This supports the experimental data, indicating that DCN neurons can adapt their population tuning frequencies to encode motor frequencies without significantly changing their intrinsic firing properties.

Taken together, the DCN neurons encode the frequencies of motor kinematics throughout populational recruitment. While each neuron generates noisy or stochastic signals, the neuronal population achieves a high signal-to-noise ratio and precise frequency coding. This confirms that LFPs, as spatiotemporal summations of these population activities, accurately reflected the synchronized frequencies between neuronal codes, LFPs, and motor kinematics.

### Rhythmic DCN stimulation induces motor rhythms.

**Fig. 4 |:**
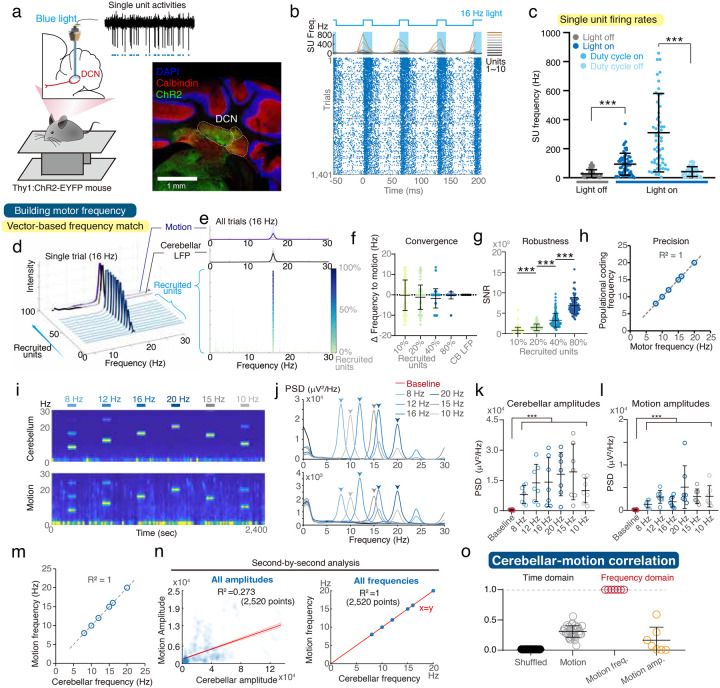
Cerebellar and motor responses to optogenetic DCN stimulation at multiple frequencies. **(A)** Schematic of the experimental setup and representative histology of channelrhodopsin-2 (ChR2)-expressing DCN. **(B)** Representative traces showing SU firing rates (top) and their modulation during 16-Hz optogenetic stimulation of the DCN (bottom). **(C)** Statistical analysis of SU firing rates across different phases of the 16-Hz stimulation cycle (n = 58 units from 6 trials in 2 mice). **(D–G)** Vector strength analysis, including a representative example (D), group analysis (E), frequency differences between motion and vector strength spectrum peaks (F), and signal-to-noise ratio of the spectrum peaks (G). **(H)** The scatter plot of peak cerebellar LFP frequencies against combined vector strength spectrum peaks under various stimulating frequencies. **(I-J)** Representative time-frequency plots (I) and spectral diagrams (J) of optogenetically driven cerebellar oscillations and corresponding motor activities. **(K-M)** Collective data from 7 trials in 3 mice showing the close correspondence between cerebellar oscillatory and motor frequencies (M). **(N)** Scatter plots of the amplitudes (left) and frequencies (right) of cerebellar LFPs and motor activity, compiled from 1-second intervals across all trials (2,520 points from 7 trials in 3 mice). **(O)** Statistical analysis of the correlation between cerebellar LFPs and motor activity in the time domain and the determination coefficient (R^2^) of the linear regression presented in Fig. 4N. Error bar denotes S.D. ***p<0.001. Kruskal-Wallis test and one-way ANOVA.

To establish the causality of the frequency-coding mechanism in motor kinematics, we optogenetically stimulated DCN neurons in *Thy1:ChR2-EYFP* mice and recorded the resultant motor kinematics using a pressure-sensing force plate^13,18^ (Fig. 4a). Rhythmic stimulation led to a periodic increase in neuronal firings (Fig. 4b). Consistently, the single-unit firing rates were way above the motor frequencies (Fig. 4c), against the rate-coding algorithm. Instead, the rhythmic optogenetic stimulation generated motor rhythm at the stimulating frequencies, and the populational tuning frequencies precisely converged to the motor frequencies at all tested scenarios (Figs. 4d–h). Phase analysis further verified the consistent feature of populational recruitment at all stimulating frequencies (**Supplementary Fig. 10**).

We also evaluated cerebellar LFPs simultaneously recorded with the motor kinematics (Fig. 4i–j). The optogenetic stimulation led to increased but varied amplitudes of cerebellar oscillatory strengths and motor rhythms (Fig. 4k–l). However, cerebellar and motor frequencies were always matched (Fig. 4m). The second-by-second analysis revealed amplitude variations across time, while the oscillatory and induced motor frequencies were always matched (Fig. 4n). Comparison between time and frequency domains confirmed that amplitude variability contributed to the imprecise cerebellar coding of rhythmic movements, while frequency information remained numerically precise (Fig. 4o).

Furthermore, a strong phase relationship between DCN firings and cerebellar LFPs indicated potential interactions between the cerebellar cortex and DCN (**Supplementary Fig. 11**). We also explored the role of axonal projections from Purkinje cells (PCs) to DCN in this frequency-coding process (**Supplementary Fig. 12**). Rhythmic stimulation of PC axonal terminals generated rhythmic motions at the stimulating frequencies with matched populational coding mechanism, phase recruitment, and cerebellar oscillations across all tested mice (**Supplementary Figs. 12–14**). Computational modeling also echoed the same results (**Supplementary Fig. 9**).

Taken together, DCN neurons generate populational tuning of firing probabilities throughout PC-to-DCN modulations.

### The cerebellum generates dynamic frequency evolution for non-rhythmic movements.

While previous results detailed the cerebellum’s encoding of rhythmic movements, most everyday movements are non-rhythmic. Theoretically, any finite signal, whether rhythmic or not, can be fully represented and reconstructed in the frequency domain. Non-rhythmic signals can be constructed using dynamically changing instantaneous phases/frequencies and amplitudes (via Hilbert transform) or multiple sets of these components in linear combinations (via Hilbert-Huang transform). Therefore, if the cerebellum can generate highly dynamic frequencies across time, it has the potential to create non-rhythmic complex motor kinematics with the same frequency coding mechanism.

**Fig. 5 |:**
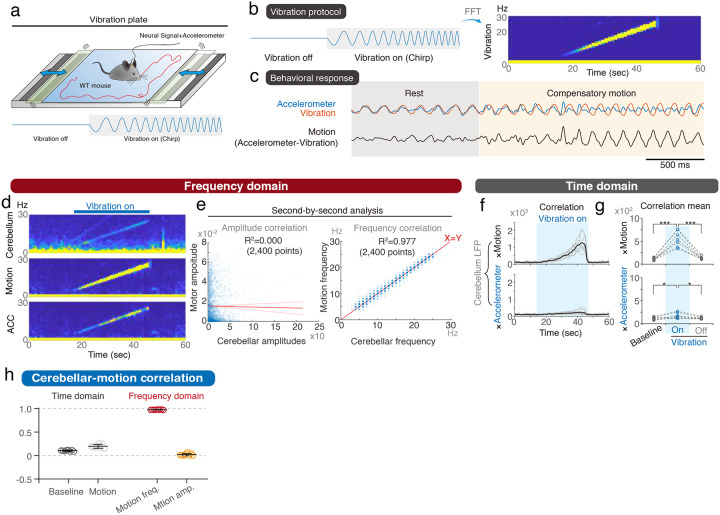
Non-rhythmic cerebellar oscillations and motor kinematics induced by linear chirp vibrations. **(a)** The experimental settings and platform vibrations with constantly changing chirp waveform. **(b)** Schematic representation of vibration protocol and the time-frequency plot of the vibration signals. **(c)** Representative traces for compensatory motions. **(d-e)** Frequency domain analysis. A representative time-frequency plot of cerebellar LFPs, motions, and accelerometer signals (ACC) (d). Linear regression analysis of second-by-second amplitudes and frequencies between the cerebellar LFPs and motions (e, 2,400 points from 80 trials in 8 mice). **(f-g)** Time domain analysis. Trial-by-trial (f) and group analysis (g) of cross-correlation for cerebellar oscillations between compensatory motions and residual body movements (accelerometer). **(h)** Statistical analysis of the correlation between cerebellar oscillation and motion in both the time domain (Pearson correlation) and the frequency domain (R^2^) (n = 8 mice). Error bars denote S.D. *p < 0.05, ***p < 0.001, One-way ANOVA.

To explore this hypothesis, we introduced floor vibrations with a linear chirp waveform to mice—a complex, non-rhythmic waveform characterized by constantly changing frequencies in a designed linear trend (Fig. 5a–c and **Video S3**). This waveform is a strictly non-rhythmic pattern in which the instantaneous frequencies at any two moments are different. Using a linear chirp vibration from 4–25 Hz in 30 seconds, the mouse cerebellum generated dynamic cerebellar oscillations and compensatory motions with matched frequency dynamics of the designed protocol (Fig. 5d–e). These self-generated cerebellar oscillations correlated strongly with compensatory motions but showed minimal correlation with residual body movements recorded by an accelerometer (Fig. 5f–g). Consistently, while frequency-dependent amplitudes of both cerebellar oscillations and motions were significantly increased (Fig. 5d), the magnitudes of increment remained poorly correlated in second-by-second analysis, therefore prohibiting the precise amplitude coding of motor kinematics (Fig. 5h).

**Fig. 6 |:**
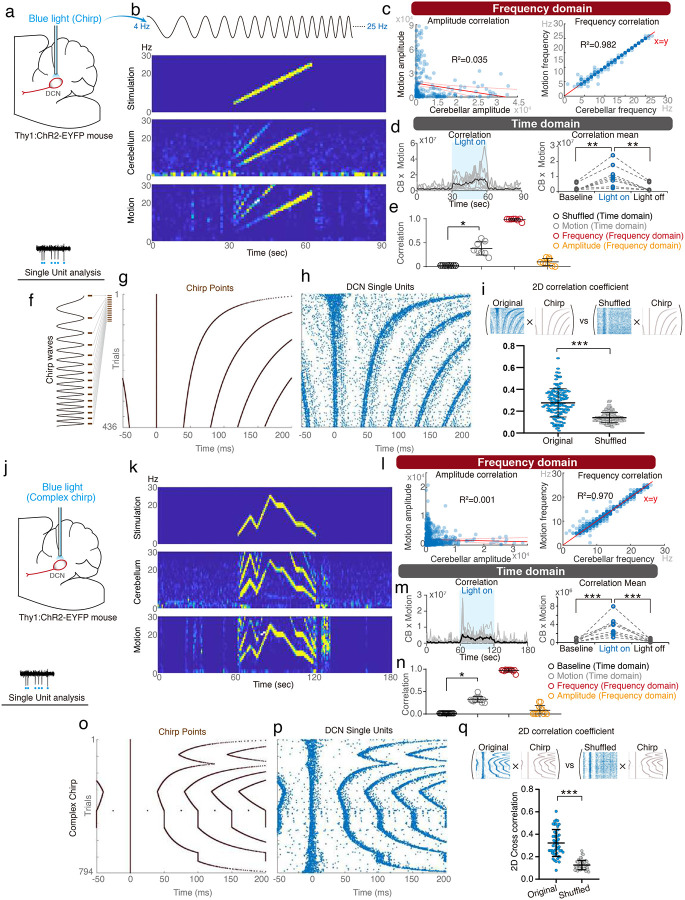
Non-rhythmic cerebellar oscillations and motor kinematics induced by optogenetic stimulation. **(a)** Optogenetic DCN stimulation with linear chirp waveform. **(b)** Representative time-frequency plot of stimulating signals, cerebellar LFPs, and motions. **(c)** Frequency domain analysis, linear regression analysis of second-by-second amplitudes and frequencies between the cerebellar LFPs and motions (239 points in 8 mice). **(d)** Time domain analysis. Trial-by-trial (left) and group analysis (right) of cross-correlation for cerebellar LFPs between motions. **(e)** Statistical analysis of the correlation between cerebellar oscillations and motions in both the time domain (Pearson correlation) and frequency domain (R^2^) (n=8 mice). **(f)** SU activities of DCN with linear chirp-wave stimulation. **(g)** Predicted chirp points of maximal firing probability and their evolution across stimulation trials (defined by the number of peaks of chirp waves). **(h)** Activity evolution of a representative SU. **(i)** Group analysis of correlation coefficient of DCN firings and chirp waveforms (n = 136 units from 8 mice). **(j-q)** Complex chirp waveform stimulation (l, 710 points in 12 mice; q, 48 units in 12 mice). Error bars denote S.D. *p < 0.05, **p<0.01, ***p < 0.001, One-way ANOVA (D, E, M, N), Wilcoxon matched-pairs signed rank test (i, q).

Next, we optogenetically illuminated the DCN with the same linear chirp in *Thy1:ChR2-EYFP* mice. Cerebellar oscillations can be reliably generated with precisely matched time-frequency dynamics. More importantly, the mice developed complex motor kinematics with the motor frequencies that matched the cerebellar oscillatory frequencies at nearly every time point (Fig. 6a–d and **Video S4**). Analysis of DCN single-unit activities during chirp stimulation revealed a unique pattern of neuronal recruitment consistent with the prediction from stimulation dynamics (Fig. 6f–i). The neurons’ ability to follow these complex temporal dynamics supports their role in forming rapidly changing frequency dynamics. Consistently, the populational DCN firing probabilities were faithfully tuned to Hilbert-based instantaneous phases/frequencies, cerebellar LFPs, and motion kinematics (**Supplementary Fig. 15**).

Besides linear chirp, we further pushed the complexity of frequency dynamics by optogenetically illuminating DCN with complex chirp waveforms (Fig. 6j–k). Like the simpler linear chirps, complex chirp illumination evoked corresponding dynamics of cerebellar oscillations (Fig. 6l–n) and neuronal firings (Fig. 6o–q), thus generating matched frequency dynamics of mouse motor kinematics. While we achieved frequency precision for simple or complex motor kinematics, the motor amplitudes remained imprecisely correlated (Figs. 6c & 6n). Therefore, this approach has yet to generate functional or skilled movements, which requires precise coding for both motor frequencies and amplitudes across all time points.

Taken together, the cerebellum reports complex frequency dynamics and matched motor kinematic frequencies in self-generated, non-rhythmic movements. Optogenetic stimulation confirmed that the cerebellum can causatively construct non-rhythmic motor kinematics by dynamically encoding motor frequencies. With the preserved algorithm and numerical precision of frequency coding across all tested mice, we can optogenetically create complicated motor kinematics with designed motor frequencies.

### Cerebellar frequency coding predicts skilled tongue movements.

The vibration platform and force plate targeted global body motions with multi-joint synchrony. we sought to determine whether the cerebellar frequency-coding algorithm could predict more localized, skilled movements. Therefore, we investigated the tongue movement during licking behaviors with simultaneous electrophysiological recordings from the dentate nucleus of DCN (**Supplementary Fig. 16a-c**). Consistently, the frequencies of dentate LFPs were highly correlated with the licking rates (**Supplementary Fig. 16d**), and the single-unit activities were tuned with the dentate LFPs at the populational level (**Supplementary Fig. 16e-h**).

Taken together, the cerebellum encodes frequency dynamics for complex motor kinematics, which is evident in global body movements and skilled tongue movements.

### The human cerebellum engages in rhythm control of volitional movements.

**Fig. 7 |:**
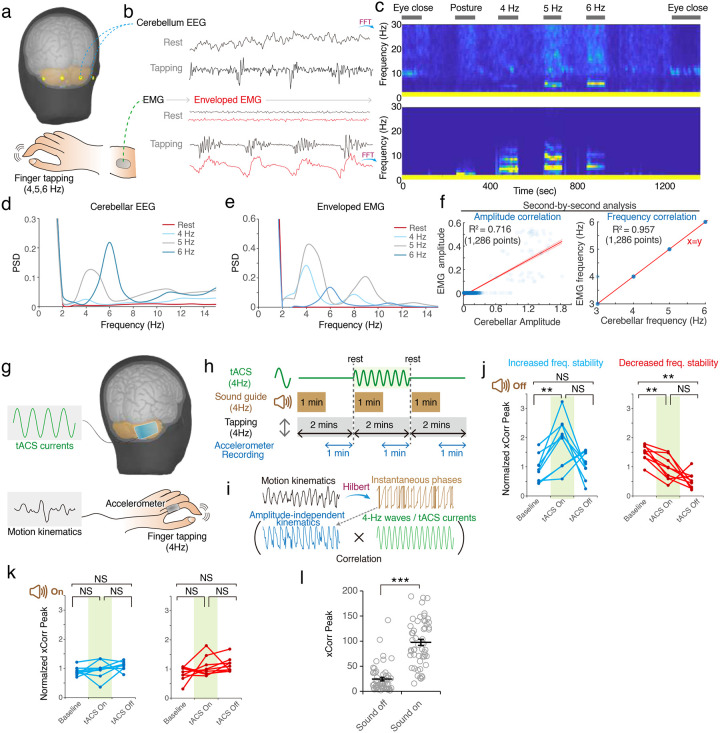
Cerebellar oscillations and their frequency modulation during volitional tapping of health subjects. **(a)** Experimental settings of cerebellar EEG and electromyography (EMG). **(b-e)** Representative traces (b), time-frequency plots (c) and, spectral diagram (d-e) of cerebellar and EMG. **(f)** Linear regression analysis of second-by-second amplitudes and frequencies of cerebellar oscillations and EMG activities at the tapping frequencies (1,286 points, *n* = 10 subjects). **(g)** Cerebellar transcranial alternating current stimulation (tACS) and simultaneous recording of tapping kinematics. **(h)** Study protocol. tACS was set at the tapping frequency of 4 Hz and applied during the middle 2 minutes of volitional tapping. **(i)** Frequency stability calculated from amplitude-independent kinematics (see methods). **(j)** tACS modulation of the frequency stability of motion kinematics without a sound guide. Bi-directional modulation was observed (*n* = 6 subjects with 3 repeated experiments; 9 and 9 trials with increased and decreased of frequency stability, respectively). **(k)** tACS modulation of the frequency stability of motion kinematics with a sound guide. No significant modulation was observed. **(l)** Cross-correlation (xCorr) peaks between tapping kinematics and tACS waveform. Values in the sound-on period were significantly higher than the sound-off period (the same 18 trials in 6 subjects). Error bars denote S.E.M.. **p < 0.01, ***p < 0.001, Wilcoxon signed-rank test.

To examine whether the human cerebellum also engages in frequency control of volitional movements, we analyzed cerebellar EEG and corresponding surface electromyographic (EMG) signals of healthy subjects performing rhythmic tapping at 4, 5, and 6 Hz (Fig. 7a–b, and **Table S1**). Mirroring our findings in mice, cerebellar oscillations were detected during finger tapping, closely matching the EMG signal frequencies in a second-by-second analysis (Fig. 7c–f).

To probe the causal role of frequency coding in the human cerebellum, we employed transcranial alternating current stimulation (tACS) to modulate cerebellar oscillations. Using strong currents to modify the frequency of cerebellar oscillations may be dangerous. Therefore, we evaluated the frequency stability of motions by applying 4-Hz tACS to healthy subjects during 4-Hz finger tapping (Fig. 7g–i and **Table S1**). Similar to the effects of bidirectional modulations of tremor amplitudes by cerebellar tACS^24^, in-phase or anti-phase stimulation may bidirectionally change the stability of motor rhythms. We utilized a 4-Hz click sound to aid subjects in adjusting their tapping frequencies and recorded accelerometer-based kinematics during both sound-on and sound-off periods. The amplitude-independent kinematics were extracted to evaluate frequency stability (see methods). During the sound-off periods, tACS was found to either increase or decrease tapping frequency stability (Fig. 7j), demonstrating effective frequency modulation. During the sound-on period, the tapping kinematics were tightly guided by the sound, therefore revealing a better correlation to the 4-Hz waveforms without a difference to tACS manipulation (Fig. 7k–l).

Taken together, the cerebellar circuit of the healthy subjects also actively engages in frequency coding of volitional movements. Manipulation of cerebellar oscillations could enhance or suppress the frequency stability of motor rhythms.

## Discussion

In this study, we provided mouse evidence and supporting human evidence that the cerebellum encodes motor frequencies for physiological motor kinematics. The frequency is encoded by the integrative phasic-tuning of neuronal firing probabilities at the populational level. While the motor amplitudes are highly variable and contribute to the variability of cerebellar kinematic coding in the time domain, the cerebellum encodes motor frequency with quantitative precision and generalizability across individuals without the need for additional calibration. This level of precision allows us to engineer frequency dynamics for complex motor kinematics in mice. Among many cerebellar functions, cerebellar rhythm coding emerges as a numerically precise and generalizable algorithm, potentially serving as a mathematical backbone for future quantitative studies of neural dynamics. The key features of frequency coding are summarized in **Supplementary Fig. 17**.

There are limitations in this study. First, the study design did not include topographical information about different muscle groups, which have been described in the cerebellum^1,25^. We applied a vibration platform for physiological global movements with multiple muscle groups activated at the same frequency. This approach enhanced the frequency-related information against the background but lost the topographical information of muscle groups. The skilled licking movements only involved tongue muscles and minimized topographical concerns. Future studies are required to demonstrate topography-based frequency coding for detailed motor kinematics. Second, we presented the human evidence that supports the causative roles of frequency modulation of the cerebellum by tACS interventions. However, we did not have the single-cell level of evidence to describe whether mouse and human cerebellar oscillations are generated based on the same mechanism of populational neuronal recruitment. Addressing this gap will likely require intra-surgical recordings or other methods capable of capturing detailed neuronal activity.

The impact of this work is to reveal the cerebellum’s use of a simple yet mathematically precise algorithm to manage the diversity and complexity of various movements. However, this is just one aspect of many cerebellar functions, such as motor learning and cognitions. Notably, frequency precision cannot be directly transferred to motor precision. For example, skilled movements like reaching, which typically involve simple trajectories and are characterized by low-frequency kinematic components, may suffer from temporal imprecision due to the inherent mathematical trade-off between spectral and temporal resolution. This issue could compromise the precision of such skilled movements. Notably, the cerebellum employs a different strategy for enhancing motor precision, specifically by fine-tuning the deceleration of movements^1,2^.

Moreover, while the olivocerebellar pathway can precisely construct dynamic motor frequencies, the counterpart mechanism for motor amplitude coding remains elusive and more complex. Our current findings (Figs. 2, 4–7) and previous studies^13,18,26^ in both mice and humans suggested that the amplitudes of frequency-dependent cerebellar oscillations significantly influence frequency-dependent motor amplitudes. Yet, variations in motor amplitudes under consistent levels of optogenetic stimulation or cerebellar oscillations indicate the presence of additional mechanisms beyond cerebellar oscillations and populational coding. Future research needs to elucidate the mechanisms responsible for encoding instantaneous amplitudes, which are crucial for constructing functional motor kinematics.

## Methods

### Animals

All experimental procedures were conducted following the guidelines and approved by the Institutional Animal Care and Use Committee (IACUC) of National Taiwan University (Protocol numbers: B201900034, B202000003, B202100150). Mice were housed in the central and satellite facilities of National Taiwan University with a reversed 12-h light/12-h dark cycle and unrestricted access to water and food. For optogenetic stimulation in the DCN, we utilized *Thy1-ChR2-EYFP* mice (B6.Cg-Tg(Thy1-COP4/EYFP)9Gfng/J, Jackson Laboratory, No. 007615), which express ChR2-EYFP in various brain region including our target DCN. For optogenetic stimulation in the PCs, we crossed *Calbindin-Cre* mice (B6;129S-*Calb1*^*tm2.1(cre)Hze*^/J, Jackson Laboratory, No. 028532) with Ai32 mice (B6;129S-*Gt(ROSA)26Sor*^tm32(CAG-COP4*H134R/EYFP)Hze^/J, Jackson Laboratory, No. 012569) mice. The resulting calbindin x Ai32 mice express channelrhodopsin-2 dominantly in PCs.

### Motion recordings in freely moving mice

Motion signals of mice were amplified and detected using a 15×22 cm force-sensitive platform (Convuls-1, Columbus Instruments), allowing the mice to move freely. The platform linearly converted the applied weight into voltage for recording, with a conversion rate of 0.45 Volts per Newton (or 141 millivolts per 32 grams of mass per gravity), enabling the platform to sense subtle weight changes caused by the mice’s motion. The data were then low-pass filtered at 250 Hz and then digitized at 1,000 Hz using a DAQ device (Cerebus, BlackRock microsystem). Detailed information regarding the systems and settings can be found in our previous paper^13^.

### Optetrode implantation and electrophysiology recording

Optetrode^27^, a combination of tetrode and optical fiber, was applied to record single unit activity, deep LFPs and perform optogenetic manipulation simultaneously. The construction of the optetrode involved threading tungsten tetrodes (California Fine Wire Company) and an optical fiber (ThorLabs, FT200UMT) through a microdrive screw (Renishaw) in a 3D-printed tower to stabilize and secure them. Each individual tungsten wire of the tetrode was threaded through the channel holes of the electrode interface board and anchored them using gold pins. Additionally, we utilized small screws (Antrin Miniature Specialties, 0.089 inches in diameter, 0.0625 inches in length) as electrodes to record the LFPs of brain surface of mice.

During the surgery, 3-month-old mice were fixed on the stereotaxic frame under anesthesia with isoflurane. The optetrodes were implanted at the DCN (AP, −6.24 mm; ML, ±2.1 mm; DV, −1.9 mm from dura), and the screws were implanted on bilateral cerebellum surface (AP, −6.24 mm; ML, ±2.1 mm). To identify the implanted trajectory of the optetrode, NeuroTrace^™^ Dil (ThermoFisher, N-22800), a tissue-labeling paste, was applied to coat the surface of the optetrode. After the implantation, we applied dental cements (Superbound, Sun Medical Co., LTD) on the skull to secure the electrodes in place at the end of the surgery.

Electrophysiology signals were sampled at a rate of 30,000 Hz using a DAQ device (Cerebus, BlackRock microsystem or Open Ephys) for subsequent offline analysis, which will be described in detail in the following sections.

### Optogenetic stimulation in the cerebellum

We utilized a custom-written LabView code to trigger the output of a diode laser (Cobolt, 473 nm) through a multifunction I/O device (NI 782258–01). This setup allowed us to precisely and linearly tune the output power at a frequency of 2 MHz. The laser power was adjusted individually for each mouse and ranged from 0.5 mW to 5 mW. To ensure accurate light power levels, daily calibrations were performed using power meters (Thorlabs) before the experiments.

In the experiments using multiple stimulating frequencies, trains of blue light (25% duty cycle) at 8, 12, 16, 20, 15 and 10 Hz were sequentially given for 90 seconds, separated by 300-second light-off periods. In the chirp stimulation experiment, linear chirp waves (30 seconds, from 4 Hz to 25 Hz) and complex chirp waves (1–10 seconds: 4–14 Hz, 10–15 seconds: 14–8 Hz, 15–25 seconds: 8–25 Hz, 25–30 seconds: 25–20 Hz, 30–35 seconds: 20 Hz, 35–45 seconds: 20–10 Hz, 45–50 seconds: 10Hz, 50–60 seconds: 10–4 Hz) were generated by MATLAB function and linearly transformed into laser power with the 30,000-Hz amplitude updating rate.

### Vibration platform

We applied a customized vibration platform with optical grating to ensure precise control of vibration frequency and its sinusoidal vibrating waveform up to 120 Hz at the amplitude of 3-mm horizontal vibrations. Two cameras were set to capture the front view and top view of the vibration platform. In the experiments using multiple vibrated frequencies, the platform vibrated at 8, 12, 16, 20, 15, and 10 Hz sequentially, with a duration of 90 seconds in each frequency and separated by 2 minutes of non-vibrating periods. In the chirp vibrated experiment, 10 times chirp vibration periods (30 seconds, from 4 Hz to 25 Hz) were separated by 30 seconds of non-vibrating periods, and we repeated the protocol for 10 times in each experimental section. We used Open Ephys acquisition board to record neural electrophysiology signals, mouse accelerating signals, and vibrated signals. Mouse accelerating and vibrated signals were captured through a headstage containing an accelerometer and an accelerometer attached to the vibration platform, respectively. The signals were recorded and digitized at the sampling rate of 30,000 Hz. To obtain the compensated motion signals, we applied a band-pass filter within the frequency range of 3–30 Hz to the vibrated signals and the mouse accelerating signals. We subtracted the mouse accelerating signals from the vibrated signals, resulting in the compensated motion signals.

### Single-unit spike sorting and burst detection

Spikes were sorted by either two sorting tools, Offline Sorter^™^ (OFS) software and Kilosort3 software^28^. Electrophysiology data acquired through optetrode were high-pass filtered at 250 Hz, and the noise were reduced through digital referencing. Offline Sorter^™^ focuses on those with higher amplitude, and extracts them as spikes. Subsequently, it performs K-means clustering to assign each extracted spike to specific single units. Kilosort3 models the electrophysiology data as a sum of template waveforms triggered on the spike times, enabling the identification and resolution of overlapping spikes. The detection criteria of DCN bursts followed previous studies (*74, 75*). The inter-spike interval within a burst should be equal or smaller than 15 ms. The minimal spike counts within a burst was 4.

### Spectrum analysis of motion and LFP data

The LFP data underwent spectrum analysis following the procedures consistent with our previously works^13,29,30^. The digitized data was down-sampled to 1,000 Hz for analysis. Frequency domain analysis was performed using in-house MATLAB scripts. Welch’s method with a Hanning window (each segment is 1-second long and overlaps half of the samples) was utilized to estimate power spectral density (PSD, μV^2^/Hz for LFP data and mV^2^/Hz for motion data). For fixed frequency stimulation, each PSD data point was calculated from a 20-second window with a 1-second shift. For chirp wave stimulation, a 1-second window without overlap was applied.

### Vector Strength Analysis

The analysis of single-unit spike timing modulation was carried out using vector strength analysis. It was introduced by Goldberg and Brown in 1969 and has been widely utilized to quantify the phase-locking and synchronization of a spike train, indicating whether a single unit fires at specific phases of a particular modulation frequency^19–23^. The spike timings of individual units were obtained using the methods discussed earlier, and these spike times, represented as a vector ***(t)***, were converted into phase angles ***(p)*** using the following formula:

p=2πft,

where f represents frequency. Phase angles were adjusted to range from −*π* to *π*. The vector strength ***(v)*** is then calculated with the equation below^31^:

$$v=\frac{1}{n}\left|\overset{n}{\underset{j=1}{\sum }}e^{ip_{j}}\right|,$$

where n is the count of spikes, p is the vector of phase angles, and i is the imaginary unit. Since a higher number of spikes often leads to a smaller vector strength, we normalized the vector strength to account for this bias^32^. We first generated a distribution of random vector strengths for n number of spikes by calculating vector strength with n random phases in 20,000 iterations. The mean and standard deviation of this distribution are then calculated, and the normalized vector strength is the original vector strength subtracting the mean and dividing the standard deviation.

The above steps only result in the vector strength at a certain frequency. In order to obtain a vector strength spectrum illustrating the frequencies at which the spike train achieves phase-locking, the aforementioned steps were repeated for each frequency ranging from 1 Hz to 50 Hz with a 0.01 Hz increase. The resulting spectrum was then subjected to the removal of exponential decay and smoothed using a Gaussian-weighted moving average. Prominent peaks with prominence larger than 1% of the mean intensity in the smoothed spectrum were subsequently identified. The prominence criteria prevent from reporting random fluctuations, and the definition of prominence is defined in MathWorks documentation page. By iterating the steps above 10 times with shuffled spike times and averaging them, we acquired the shuffled vector strength that served as a control.

To assess the contribution of population coding, we summed the normalized vector strength spectra from individual units in a random sequence one by one. This resulted in a cumulative spectrum and we examined the signal-to-noise ratio (SNR) from the inclusion of 10%, 20%, 40%, and 80% of the total units. The SNR is defined as follow:

$$SNR=\frac{\text{mean}{\left(\text{siganl}\right)}^{2}}{std{\left(\text{noise}\right)}^{2}},$$

The range of “noise” pertains to a bandwidth of 5 Hz characterized by the least intensity. To mitigate potential bias, this iterative procedure was replicated 100 times. All these procedures were executed using an in-house MATLAB script.

### Correlation spectrum (autocorrelogram)

We conducted an analysis of the firing modulation of single units to assess their periodic activity. The single-unit data was down-sampled from 30,000 Hz to 250 Hz and subsequently binarized into an array containing either 0 (indicating time without spike firing) or 1 (indicating time with spike firing). This binary array underwent autocorrelation using a maximum lag of 1 second, resulting in an autocorrelation function (ACF). To determine the firing modulation of the single unit, we applied the fast Fourier transform (FFT) to the ACF with a frequency resolution of 0.1 Hz. We extracted significant frequency components by identifying peaks in the frequency spectrum of the firing modulation, with a prominence exceeding 1% of the mean intensity. As with our vector strength analysis, our examination focused on the frequency range of 0–30 Hz, which corresponds to linear motor kinematic coding. We replicated the random-recruiting approach on the vector strength spectrum. The definition of the signal-to-noise ratio remained consistent. All the procedures detailed above were implemented using an in-house MATLAB script.

### Spike-phase analysis

To examine the phasic tuning relationship between the single unit firing probability and the continuous data (cerebellar LFP and the motor kinematics), we coupled the single-unit spikes time with the instantaneous phase of the continuous data. First, both the single-unit spikes time and the LFP were down-sampled from 30,000 Hz to 1,000 Hz to facilitate effective filtering. Next, we applied a band-pass filter to the continuous data with a range of ± 3 Hz around the frequency of interest (e.g., 4, 8, 12, 16, 20, 15 and 10 Hz). Utilizing the Hilbert transform, we calculated the instantaneous phase of the filtered data and corrected it by π/2. Extracting the phase corresponding to each single unit spike time, we visualized these extracted phases as polar histograms. Furthermore, we introduced a control by shuffling the instantaneous phases and pairing these randomized phases with each spike time, resulting in shuffled polar histograms. To quantify the phasic bias, we computed the polarity index^13^. This index involves summing each phase as a unit vector and then dividing by the total number of vectors. The polarity index ranges between 0 (indicating a purely random distribution across phases) to 1 (indicating a completely biased distribution towards a specific phase).

### Correlation analysis of cerebellar LFP data

To examine the relationship between cerebellar LFP and various signals (vibrated signals, accelerating signals, motion signals, and chirp stimulation signals of laser), we calculated their cross-correlation using an in-house MATLAB script based on the “xcorr()” function. The cross-correlation was computed with a 1-second window that shifted along the data. We extracted the maximal values from each calculation, resulting in a time series of the maximal cross-correlation between cerebellar LFP and the other signals.

### 2D correlation analysis of chirp stimulation pattern

An ideal stimulation pattern generated from the chirp wave mentioned previously was obtained by aligning each stimulation point at 0 and plotting all stimulation points from −50 ms to 200 ms. The evoked potential of DCN single units were aligned and plotted in the same way, resulting in 2D binary matrices of the same dimension. The 2D correlation coefficient between the ideal stimulation pattern and the experimental results was calculated, producing a single value indicating the similarity between the patterns. Shuffled patterns were generated by permutating the timepoints of DCN single-unit spikes. All the steps mentioned above were achieved by in-house MATLAB script.

### Computational simulation

#### The neuron model.

We used the leaky integrate-and-fire model as described previously^33–35^. In the model, the membrane potential *V* of a neuron is given by

$$C\frac{dV}{dt}=-g_{L}\left(V-V_{L}\right)-g_{s}s\left(V-V_{s}\right)+I,$$

where *C* is the membrane capacitance, *g*_*L*_ is the membrane leak conductance, *V*_*L*_ is the membrane resting potential, *g*_*s*_ is the synaptic conductance, *s* is the synaptic gating variable, *V*_*s*_ is the synaptic reverse potential and *I* is other input currents. We further simplified the model into the following equivalent form by dividing both sides by *C*, which leads to

$$\frac{dV}{dt}=-\frac{1}{\tau }\left(\left(V-V_{L}\right)+g_{s}^{\prime }s\left(V-V_{s}\right)-i\right).$$

The conductance on the right-hand side of the equation is absorbed into 1C. As a result, $g_{s}^{\prime }$ is a unitless variable, and the input *i* has the unit of voltage. In the model, we also added Gaussian noise as the membrane current, which is given by $\sqrt{\tau }\sigma \chi$, where *χ* is a Gaussian distributed noise with zero mean and unit standard deviation *σ* describes the magnitude of the noise. Adding the noise term into the equation above leads to

$$\frac{dV}{dt}=-\frac{1}{\tau }\left(\left(V-V_{L}\right)+g_{s}^{\prime }s\left(V-V_{s}\right)-i\right)+\frac{\sigma \chi }{\sqrt{\tau }}.$$

The gating variable *s* is given by

$$\frac{ds}{dt}=-\frac{s}{\tau_{s}}+\sum_{k}\delta \left(t-t_{k}\right),$$

where *τ*_*s*_ is the synaptic time constant and *t*_*k*_ is the time of *k*-th input spike. The delta function *δ*(*x*) is ∞ at *x* = 0 and 0 elsewhere. We modeled the excitatory and inhibitory (GABAergic) synapses. The time constant (*τ*_*s*_) equals 2ms for both types of synapses, and the reverse potential (*V*_*s*_) is 0mV for the excitatory and −70mV for the inhibitory synapses.

#### The network model.

The network contains two neural populations, PC (Purkinje cells) and DCN (deep cerebellar nucleus), and each population contains 100 neurons. Each PC neuron receives a noise input (*σ* = 10 *mV*) and a sinusoidal input *i* = *A* sin(2*πft*) with amplitude: *A* = 80 mV and modulatory frequency: *f* = 16Hz. The PC neurons project to the DCN neurons with one-to-one connections via GABAeregic synapses ($g_{s}^{\prime }=0.7$). The DCN neurons are known to exhibit spontaneous activity, which is modeled by applying a constant membrane current (*i* = 20*mV*) and a Poisson spike train (100 Hz) through the excitatory synapse ($g_{s}^{\prime }=0.3$) to each DCN neuron. These inputs elicit a spontaneous firing rate of about 20–22 Hz in each DCN neuron.

#### LFPs.

The LFPs of DCN are derived by calculating the mean EPSC (excitatory postsynaptic current) and mean IPSC (inhibitory postsynaptic current) across all DCN neurons and then taking the average of the two mean currents. The EPSC contributes to the negative component of the LFP, while the IPSC contributes to the positive component of the LFPs^36^. We did not consider the distance factor of the neuron in relation to its contribution to the LFPs because we only modeled 100 DCN neurons, and no topographical correlation between these neurons was assumed.

#### The simulation protocol.

We performed a 20,000 ms simulation in each trial. The first 5,000 ms was the resting period in which no sinusoidal input to the PC neurons was provided. PC neurons generally did not fire without the sinusoidal input. Therefore, the DCN neurons were not driven by the PC neurons and only exhibited spontaneous firing activity. After resting, the trial entered a 10,000 ms stimulation period in which the sinusoidal input to the PC neurons was turned on. After the stimulation period, the sinusoidal input was removed, and the trial entered a 5,000 post-stimulation resting period. The spike times EPSC and IPSC of all DCN neurons were recorded during the trial.

#### The data analysis.

We calculated the power spectrum density of LFP and analyzed the vector strength of the spike trains of the DCN neurons using methods similar to those described in the Methods section of the main text. The LFP spectrum was calculated using Welch’s method with a Hanning window of 1s. The vector strength was calculated for different numbers of recruited units (neurons) to reveal the effect of population coding. The vector strength was normalized by subtracting the mean and then divided by the standard deviation of the random baseline data, which was calculated based on the vector strengths of 1,000 randomized spike trains.

### Tissue clearing and histological validation

After completing the behavioural experiments, mice were perfused transcardially with 4% paraformaldehyde. Their brains were retrieved for further examination of electrode placement and fluorescent expression. Coronal or sagittal sections of were cut with a thickness of 500 μm using vibratome, and underwent tissue clearing with RapidClear (Bio-East technology) for 1 week. The histology images were acquired with fluorescent confocal microscope (SP8, Leica). We assessed both the electrode placement and the fluorescent expression pattern of *Thy1-ChR2-EYFP* and calbindin x Ai32. In cases where improper electrode placement or insufficient fluorescent expression was observed, the corresponding electrophysiology data from those mice were excluded from further analysis.

### Human subjects

10 healthy subjects received cerebellar EEG recordings during volitional tapping, and 6 healthy subjects received the tACS study. We recruited these subjects from two institutions: the Neurological Institute at Columbia University Irving Medical Center, New York, USA, and the Cerebellar Research Center at National Taiwan University Hospital, Yun-Lin Branch, Yun-Lin, Taiwan. Before participating in the study, all subjects provided written consent. The research protocols were approved by the Institute Review Board at both Columbia University and National Taiwan University Hospital. Further detailed information about the demographic of the subjects can be referred to **Table S1**.

### Cerebellar EEG recordings and analysis for healthy subjects performing volitional tapping

The cerebellar EEG recordings were also performed with the same lead settings as our previous works^13,26,37^. In healthy subjects performing volitional tapping, the EEG signals were sampled at 512 Hz with a 64-channel EEG machine (Quantum, Natus Medical Inc.). The signals also received band-passed filter at 0.3~128 Hz. Muscle activities were recorded by surface EMG, also sampling at 512 Hz by the same EEG machine and band-pass filtered between 20–128 Hz. Surface EMG data were then enveloped based on the 20-millisecond of root-mean-squared value by in-house MATLAB function. The pre-processed EEG and enveloped EMG data then underwent the same spectrum analysis described in the previous section.

### Accelerometer measurements and transcranial alternating current stimulation (tACS)

In tACS experiments, the acceleration of finger tapping and EEG were recorded using the Brain Vision acceleration sensor MR (3D) and the actiCHamp system (Brain Vision LLC, Morrisville, NC, USA). To perform tACS, we utilized a Soterix Medical 1×1 tES mini-CT device to generate a stimulated waveform, which was then delivered using two 5 × 5 cm SNAPpad sponge (Soterix Medical Inc., Woodbridge, NJ, USA) consisting pre-inserted carbon-rubber electrode, at an intensity of 2.5 mA. These sponge electrodes were firmly secured in place using a head and arm SNAPstrap. The stimulation electrode was targeted 2 cm lateral to the inion, covering the right cerebellar hemisphere, while the reference electrode was positioned on the deltoid muscles of the right arm.

The experiment involved a sound-guided, rhythmic tapping task using the index finger. Baseline recording involved 2 minutes of tapping, including 1 minute of tapping with 4 Hz guided audio sound, and 1 minute of tapping without any audio. After a short rest interval, tACS was delivered for 2 minutes during the tapping task at 4 Hz. The audio cue was applied for 1 minute in every tapping period and then turned off. After stimulation and a rest interval, the tapping task was repeated and recorded again for 2 minutes, including 1 minute of tapping with guided audio sound and 1 minute without any audio.

To assess the phase stability between the accelerometer-recorded motion and tACS, we applied the phase-sensitive cross-correlation. We transformed the motion while preserving its frequency dynamics and eliminating amplitude fluctuations by extracting their Hilbert-based instantaneous phases and replacing with the time-dependent phases of a unit vector. The transformed motion was then cross-correlated with the 4-Hz sine waves, to evaluate the rhythmicity between 4-Hz tapping and a perfect 4-Hz signals. The maximal cross-correlation values were calculated. To ensure fair comparisons among subjects, we normalized the mean cross-correlation values (averages of cross-correlation values across the entire experiment) to 1.

### Statistics

Non-parametric analyses were conducted for datasets with sample sizes below 35 or those not following a normal distribution. We applied the Mann-Whitney U test, Wilcoxon signed-rank test, and Kruskal-Wallis test for independent samples, paired groups, and multiple groups, respectively. For datasets with sample sizes exceeding 35 and meeting the homogeneity test for normal distribution, student’s T-test, paired T-test, and one-way ANOVA were employed for independent samples, paired samples, and multiple groups, respectively. Raw data points were illustrated in the figures.

## Data Availability

All data are available in the main text or the supplementary materials. Programs for data analysis are deposited in GitHub repository: https://gitfront.io/r/user-5686195/pPu4TP21pz4V/CB-Frequency-Coding-of-Motor-Kinematics/. Further details are upon request.

## Highlights

1. The cerebellum encodes motor frequencies via integrating neuronal firing probabilities at the populational level.
2. The motor frequencies can be highly dynamic to construct both rhythmic and non-rhythmic movements.
3. The causality of the frequency-coding mechanism can be validated by optogenetic manipulation in mice and current stimulations in humans.
4. The cerebellar codes for motor frequencies are mathematically precise with cross-individual uniformity.
